# Emerging role of RNA methyltransferase METTL3 in gastrointestinal cancer

**DOI:** 10.1186/s13045-020-00895-1

**Published:** 2020-05-19

**Authors:** Qiang Wang, Wei Geng, Huimin Guo, Zhangding Wang, Kaiyue Xu, Chen Chen, Shouyu Wang

**Affiliations:** 1 Department of Hepatobiliary Surgery, The Affiliated Drum Tower Hospital of Nanjing University Medical School, Nanjing, Jiangsu Province China; 2 The Affiliated Yancheng No. 1 People’s Hospital of Nanjing University Medical School, Yancheng, Jiangsu Province China; 3 Department of Gastroenterology, The Affiliated Drum Tower Hospital of Nanjing University Medical School, Nanjing, Jiangsu Province China; 4 Department of Radiotherapy, The First Affiliated Hospital of Nanjing Medical University, Nanjing, Jiangsu Province China; 5 Department of Molecular Cell Biology and Toxicology, Key Laboratory of Modern Toxicology of Ministry of Education, School of Public Health, Nanjing Medical University, Nanjing, Jiangsu Province China; 6 Jiangsu Key Laboratory of Molecular Medicine, Medical School of Nanjing University, Nanjing, Jiangsu Province China; 7 Center for Public Health Research, Medical School of Nanjing University, Nanjing, Jiangsu Province China

**Keywords:** Gastrointestinal cancer, Epigenetics, METTL3, Oncogene, m^6^A

## Abstract

Gastrointestinal cancer, the most common solid tumor, has a poor prognosis. With the development of high-throughput sequencing and detection technology, recent studies have suggested that many chemical modifications of human RNA are involved in the development of human diseases, including cancer. m^6^A, the most abundant modification, was revealed to participate in a series of aspects of cancer progression. Recent evidence has shown that methyltransferase-like 3 (METTL3), the first identified and a critical methyltransferase, catalyzes m^6^A methylation on mRNA or non-coding RNA in mammals, affecting RNA metabolism. Abnormal m^6^A levels caused by METTL3 have been reported to be involved in different aspects of cancer development, including proliferation, apoptosis, and metastasis. In this review, we will shed light on recent findings regarding the biological function of METTL3 in gastrointestinal cancer and discuss future research directions and potential clinical applications of METTL3 for gastrointestinal cancer.

## Background

**Fig. 1:**
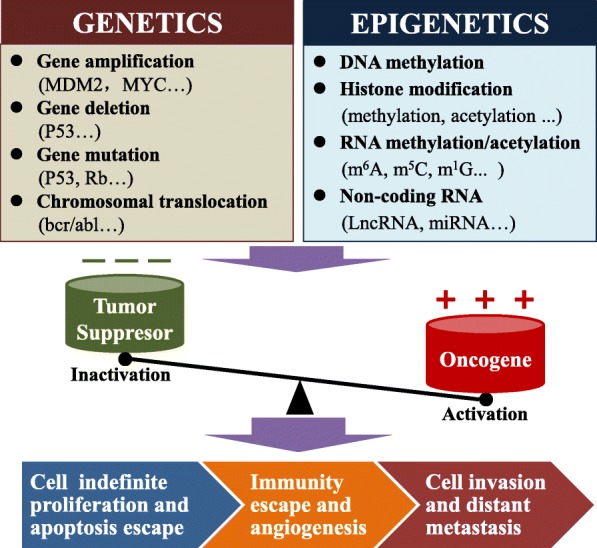
Abnormal genetics and epigenetics contribute to cancer development. Genetic changes mainly include gene amplification, deletion, mutation, and chromosomal translocation, while epigenetic changes include DNA methylation, histone modification (methylation, acetylation, etc.), non-coding RNA, and RNA methylation/acetylation. Abnormal genetics and epigenetics lead to oncogene activation and tumor suppressor gene inactivation, which result in uncontrolled cell growth and decreased apoptosis. With the development of cancer, tumor cells evade the immune system and promote angiogenesis; tumor cells can also invade the stroma via migration and invasion and enter the lymph vessels or blood vessels to cause distant metastasis

It is well known that cancer is a multistage genetic and epigenetic disease with a complex etiology involving mutation, upregulation, downregulation, and deletion of oncogenes and tumor suppressor genes [1–4]. Gene amplification/deletion/mutation or chromosomal translocation is abnormal genetic changes that lead to tumorigenesis and tumor development [5, 6]. Recently, there has been increasing evidence that epigenetic regulation plays a major role in cancer [7, 8]. Epigenetic modifications are heritable and reversible and can regulate gene expression and cancer progression without DNA sequence changes [1, 5]. Previous studies mostly focused on the role of DNA methylation, histone modification (methylation and acetylation), and non-coding RNAs in the biological function of cancer [9–11] (Fig. 1).

**Fig. 2:**
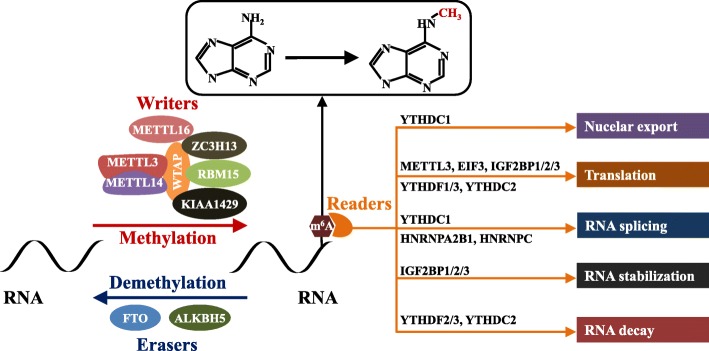
Summary of the m^6^A modification mechanism mediated by writers, erasers, and readers. The methyltransferase complex composed of the METTL3-METTL14-WTAP core component and other regulatory cofactors (KIAA1429, RBM15, ZC3H13, and METTL16) catalyses methylation at the N^6^ adenosine. Moreover, m^6^A can be reversibly removed by m^6^A eraser proteins (FTO and ALKBH5). m^6^A can also be recognized by m^6^A-binding proteins to affect mRNA fate. YTHDC1 can affect the exportation of m^6^A-modified mRNA transcripts from the nucleus to the cytoplasm, while METTL3, EIF3, IGF2BP1/2/3, YTHDF1/3, and YTHDC2 can promote the translation of RNA. YTHDC1, HNRNPA2B1, and HNRNPC can promote RNA splicing. IGF2BP1/2/3 can enhance RNA stability, while YTHDF2/3 and YTHDC2 accelerate the decay of RNA

Similar to DNA/histone modifications, more than 100 chemical modifications have been found in human RNA, which has become a hot research topic in the biological sciences and extends to a novel field of RNA modification-mediated epigenetic regulation [12–15] (Fig. 1). RNA methylation is the main chemical modification of human RNA; the types of RNA methylation include 5-methylcytosine (m^5^C), 1-methylguanosine (m^1^G), m^2^G, m^6^G, m^7^G, N6-methyladenosine (m^6^A), and m^1^A [13]**.** m^6^A modification is the most abundant RNA modification and ubiquitously occurs in eukaryotic RNA [16, 17]. m^6^A modifications account for approximately 0.1–0.4% of adenosine molecules in the isolated RNA from mammals [18]. m^6^A modification can regulate RNA stability, splicing, transport, localization, or translation and has been reported to play a critical role in different diseases, including cancer [19]. Most m^6^A sites are found within the consensus sequence RRm^6^ACH (*R* = G or A, *H* = A, C, or U) [20, 21]. m^6^A modification is reversible and dynamic in mammalian cells and can be installed by m^6^A methyltransferases (writers) and removed by m^6^A demethylases (erasers). In addition, specific RNA-binding proteins, also called readers, can recognize and bind to the m^6^A motif and influence RNA metabolism processes, including RNA stabilization, decay, splicing, translation, and nuclear export [21, 22] (Fig. 2). To date, an increasing number of novel multiple m^6^A regulatory enzymes (writers, erasers, and readers) have been identified to be involved in the regulation of m^6^A [20].

m^6^A methyltransferases are multicomponent methyltransferase complexes that consist of at least 7 “writer” proteins, including methyltransferase-like 3/14/16 (METTL3/14/16), WT1-associated protein (WTAP), vir-like m^6^A methyltransferase-associated (VIRMA, also called KIAA1429), zinc finger CCCH-type containing 13 (ZC3H13), and RNA-binding motif protein 15 (RBM15) [21, 23]. Among the complexes, METTL3 is the sole catalytic subunit that binds to the methyl donor *S*-adenosylmethionine (SAM) and catalyzes methyl group transfer [23]. In addition to METTL3, METTL14 serves as a pseudomethyltransferase to support METTL3 and recognizes target RNAs, while WTAP ensures the localization of the METTL3-METTL14 heterodimer to the nuclear speckle and promotes catalytic activity [24, 25]. METTL16 catalyzes m^6^A modification in U6-snRNA and participates in pre-RNA splicing, while RBM15 binds the m^6^A complex and recruits it to special RNA sites [26, 27]. ZC3H13 can enhance m^6^A by bridging WTAP to the mRNA-binding factor Nito [28, 29]. KIAA1429 directs m^6^A in the 3′-UTR and near the stop codon by recruiting the methyltransferase complex to modulate region-selective methylation [30]. Moreover, RNA m^6^A modification could also be removed by the 2 demethylases, including alpha-ketoglutarate-dependent dioxygenase (FTO) and alkB homolog 5 RNA demethylase (ALKBH5), thus conferring reversible and dynamic regulation of m^6^A methylation [23] (Fig. 2).

Among the m^6^A methyltransferases, METTL3 was first identified and acts as the major catalytic enzyme (writer) to catalyze m^6^A modification on mRNA and non-coding RNA [31, 32]. In recent years, the biological functions of METTL3 have been widely studied to be involved in various types of cancer development, including gastric cancer (GC), colorectal cancer (CRC), liver cancer (LC), and pancreatic cancer (PC). In the present review, we will focus on the functional role of METTL3 in gastrointestinal cancer identified by the recent findings of our and other laboratories and discuss directions for future research and potential clinical application of METTL3 for gastrointestinal cancer.

### The role of METTL3 in gastrointestinal cancer

**Fig. 3:**
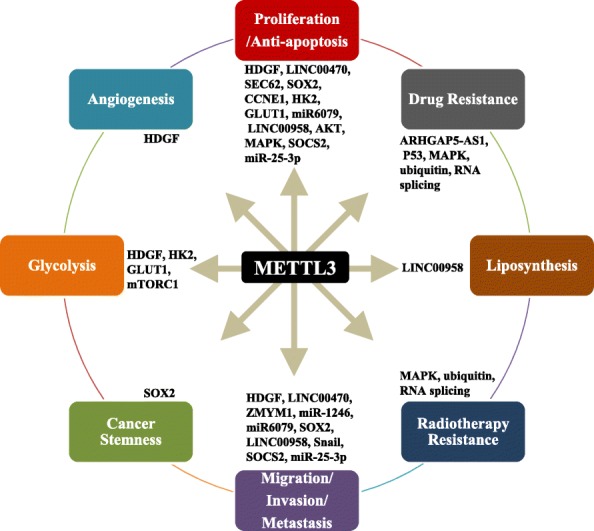
The biological function of METTL3 in gastrointestinal cancer. METTL3 regulates the differential expression of oncogenes and tumor suppressor genes at posttranscriptional levels by mediating RNA stability and translation, which contributes to processes involved in the development of gastrointestinal cancer, including cell proliferation, apoptosis, invasion, migration, metastasis, angiogenesis, radiochemotherapy resistance, glycolysis/lipid metabolism, and CSC maintenance in gastrointestinal cancer

Recent studies have shown that METTL3 is closely associated with the processes involved in the progression of gastrointestinal cancer, including tumor proliferation, apoptosis, metastasis, angiogenesis, chemo/radiotherapy resistance, glycolipid metabolism, and cancer stem cell (CSC) maintenance (Fig. 3). We herein present a summary of the recent findings of METTL3 in gastrointestinal cancer (Table 1).

**Table 1:** Role of METTL3 in gastrointestinal cancer

Cancer type	Role of METTL3	Target	Biological function	Mechanism	Upstream	Reader	Ref
GC	Oncogene	HDGF	Promotes cell proliferation, invasion, and migration, tumor growth, angiogenesis, liver metastasis, glycolysis	Enhance HDGF mRNA stability	H3K27ac	IGF2BP3	[33]
	Oncogene	LINC00470	Promotes cell proliferation, invasion, and migration	Interacts with LINC00470 to suppress PTEN stability	No study	YTHDF2	[34]
	Oncogene	ZMYM1	Promotes EMT process and metastasis	Enhance ZMYM1 mRNA stability	No study	ELAVL1	[35]
	Oncogene	SEC62	Promotes cell proliferation and inhibits apoptosis	Enhance SEC62 mRNA stability	MiR-4429	IGF2BP1	[36]
	Oncogene	EMT markers	Promotes cell proliferation, invasion, and migration	Promotes EMT process	Transcription factor GFI- 1	No study	[37]
	Oncogene	ARHGAP5-AS1	Promotes chemoresistance	Stimulates m^6^A of ARHGAP5 mRNA to enhance ARHGAP5-AS1 stability	ARHGAP5-AS1	No study	[38]
	Oncogene	AKT pathway, apoptosis pathway	Promotes cell proliferation, migration, and invasion	Activates AKT and decreases apoptosis pathway	No study	No study	[39]
	Oncogene	MYC	Promotes cell proliferation, migration, and invasion	Activates MYC	No study	No study	[40]
CRC	Oncogene	miR-1246	Promotes cell migration, invasion and metastasis	Promotes the maturation of pri-miR-1246	No study	No study	[41]
	Tumor suppressor	p-p38 and p-ERK	Inhibits proliferation, migration, and invasion	Inhibits p-p38 and p-ERK pathway	No study	No study	[42]
	Oncogene	SOX2	Promotes self-renewal, stem cell frequency, migration, and tumorigenesis and metastasis	Prevents SOX2 mRNA degradation	No study	IGF2BP2	[43]
	Oncogene	P53	Acquires multidrug resistance	Promotes pre-mRNA splicing	No study	No study	[44]
	Oncogene	CCNE1	Promotes cell proliferation	stabilizes CCNE1 mRNA	No study	No study	[45]
	Oncogene	HK2 and GLUT1	Promotes glycolysis and tumorigenesis.	Stabilized HK2 and GLUT1 mRNA	No study	IGF2BP2/3	[46]
LC	Oncogene	miR6079	accelerates progression of liver cancer cells	Promotes miR6079 expression	miR24-2	No study	[47]
	Oncogene	LINC00958	Promotes HCC cell proliferation, motility, lipogenesis, and tumor growth, metastasis	Promotes LINC00958 RNA transcript stability	No study	No study	[48]
	Oncogene	Snail	Promotes HCC migration, invasion and EMT of cancer cells both in vitro and in vivo.	Triggers polysome-mediated translation of Snail mRNA	No study	YTHDF1	[49]
	Oncogene	SOCS2	Promotes HCC proliferation, migration, colony formation, tumorigenicity, and lung metastasis	Reduces SOCS2 mRNA expression	No study	YTHDF2	[50]
	Oncogene	mTORC1	Promotes HCC glycolysis	Increases mTORC1 activity	No study	No study	[51]
PC	Oncogene	Unspecific	Promotes proliferation, invasion, and migration	No study	No study	No study	[52]
	Oncogene	miR-25-3p	Promotes tumorigenesis	Promote miR-25 processing and maturation	Hypomethylation	No study	[53]
	Oncogene	MAPK, ubiquitin, and RNA splicing	Promotes chemo- and radioresistance	Activates MAPK, ubiquitin, and RNA splicing pathway	No study	No study	[54]

*GC* gastric cancer, *CRC* colorectal cancer, *LC* liver cancer, *HCC* hepatocellular carcinoma, *PC* pancreatic cancer

### The role of METTL3 in the proliferation and apoptosis of gastrointestinal cancer

The basic characteristics of cancer include the ability to proliferate indefinitely and evade apoptosis, which are the hallmarks of cancer [55]. Many studies have demonstrated that METTL3 promotes cell proliferation and inhibits apoptosis in gastrointestinal cancer by regulating several different targets or pathways, including mRNAs and non-coding RNAs [56]. Our study showed that METTL3 protein levels were significantly upregulated in GC, contributing to poor prognosis [33]. In addition, overexpression of METTL3 accelerated GC cell proliferation both in vitro and in vivo. Furthermore, we confirmed that elevated METTL3 promoted cell proliferation using a GC organoid model. Mechanistically, METTL3 promotes m^6^A methylation on HDGF mRNA, and the reader insulin-like growth factor 2 mRNA-binding protein 3 (IGF2BP3) directly binds to the m^6^A site and enhances hepatoma-derived growth factor (HDGF) mRNA stability. Further, secreted HDGF promotes tumor angiogenesis, while nuclear HDGF activates glycolysis-related proteins, including enolase 2 (ENO2) and solute carrier family 2 member 4 (GLUT4), followed by an increase in glycolysis to cause tumor growth in GC [33]. Other studies also showed that METTL3 promotes GC cell proliferation and inhibits apoptosis through alterations of other targets and pathways, including an increase in preprotein translocation factor (SEC62) mRNA stability [36] and the activation of the AKT/MYC-related pathway [39, 40]. In addition to regulating mRNA, METTL3 also influences non-coding RNA metabolism in GC. For example, METTL3 interacts with the non-coding RNA LINC00470 to suppress phosphatase and tensin homolog (PTEN) mRNA stability, resulting in GC cell proliferation [34]. Recent findings showed that METTL3 expression was higher in CRC tissues than in normal tissues and that this feature indicated poor prognosis; upregulation of METTL3 promoted CRC tumor growth by stabilizing SRY-box 2 (SOX2) [43] and cyclin E1 (CCNE1) mRNA in an m^6^A-dependent manner [45]. However, another study showed that METTL3 was a tumor suppressor that inhibited CRC cell proliferation [42]. In human hepatocellular carcinoma (HCC), METTL3 was found to be significantly upregulated and contributed to the poor prognosis of HCC patients [50]. Functionally, knockdown or knockout of METTL3 inhibited HCC growth, while the opposite result was observed when METTL3 was overexpressed. Mechanistically, METTL3 inhibited suppressor of cytokine signaling 2 (SOCS2) expression via m^6^A-YTHDF2-dependent mRNA degradation. In addition to regulating mRNA, METTL3 also promoted HCC cell proliferation by enhancing miR-6079 expression or LINC00958 transcript stability [48]. METTL3 was also an oncogene that promoted PC cell proliferation by accelerating miR-25 processing and maturation [53]. In summary, substantial evidence has revealed that METTL3 is an independent prognostic factor for gastrointestinal cancer and that METTL3 is essential for the proliferation of gastrointestinal cancer as it regulates the stability, degradation, and maturation of mRNA or non-coding RNA.

### The role of METTL3 in the migration, invasion, and metastasis of gastrointestinal cancer

One of the main hallmarks of cancer is the activation of invasion and metastasis [55]. Metastasis is responsible for more than 90% of cancer-related deaths related to solid tumors [57]. In our study [33], overexpression of METTL3 promoted GC cell migration and invasion in vitro and liver metastasis in vivo through enhancing the stability of HDGF mRNA. Others also confirmed that METTL3 promoted the migration and invasion of GC by targeting the MYC-related pathway [40] and interacting with LINC00470 to suppress PTEN mRNA in GC [34]. Tumor epithelial-mesenchymal transition (EMT) refers to the process by which epithelial cells lose polarity, close connections, and cell-cell adhesion properties and acquire infiltration and migration abilities, which leads cells to adopt interstitial cell morphology and characteristics; this is an early and crucial step in metastasis progression [58]. Recent findings showed that METTL3 was required for the EMT process in vitro and for metastasis in vivo as METTL3 enhances zinc finger MYM-type containing 1 (ZMYM1) mRNA stability [35, 37]. In HCC, METTL3 promoted EMT and metastasis through triggering polysome-mediated translation of Snail family transcriptional repressor (Snail) mRNA [49]. In addition, knockdown of METTL3 inactivated the AKT pathway to reduce GC cell migration and invasion [39], and upregulation of METTL3 facilitated metastasis of CRC via the miR-1246/SPRED2/MAPK pathway [41] and the stabilization of SOX2 expression [43]. However, Deng et al. showed that METTL3 suppressed CRC cell migration via p38/ERK pathways [42]. In HCC, METTL3 also accelerated HCC cell metastasis by promoting the stability of the oncogenic non-coding LINC00958 RNA transcript [48] and reducing the mRNA expression of tumor suppressor SOCS2 [50]. A recent study also revealed that METTL3 promoted cell invasion and migration in PC [52], but the mechanism still needs to be further studied. Collectively, these findings reveal that METTL3 could serve as an oncogene in the EMT and metastasis of gastrointestinal cancer.

### The role of METTL3 in angiogenesis of gastrointestinal cancer

Angiogenesis is one of the most basic factors in tumor growth and metastasis and can provide nutrition for tumor tissue metabolism. In our study [33], we found that microvessel density was significantly higher in tumor tissues with high METTL3 expression than in those with low expression. In vitro, we found that the upregulation of METTL3 promoted human umbilical vein endothelial cell (HUVEC) growth and tube formation via the secretion of HDGF. Our results indicated that METTL3 may promote GC growth and metastasis by promoting angiogenesis. However, whether METTL3 is involved in the angiogenesis of other gastrointestinal cancers and thus affects the malignant process of tumors still needs further study.

### The role of METTL3 in chemo- and radiotherapy resistance of gastrointestinal cancer

Chemotherapy and radiotherapy are widely used in the treatment of solid tumors [59]. However, resistance to chemotherapy and radiotherapy due to a series of genetic and epigenetic alterations limits its efficacy [59]. A recent study revealed that lncRNA ARHGAP5-AS1 was significantly increased in chemoresistant GC cells, which contributed to chemoresistance. Furthermore, ARHGAP5-AS1 stabilized ARHGAP5 mRNA by recruiting METTL3 to stimulate m^6^A modification of ARHGAP5 mRNA and contribute to drug resistance, indicating that METTL3 was involved in chemotherapy resistance in GC [38]. In addition, the upregulation of METTL3 promoted preferential pre-mRNA splicing to produce the p53 R273H mutant protein and resulted in acquired multidrug resistance in CRC [44]. In PC, overexpression of METTL3 also contributed chemo- and radioresistance via activation of the MAPK, ubiquitin, and RNA splicing pathways [54]. These observations suggest that METTL3 is involved in chemoradiotherapy resistance in gastrointestinal cancer, indicating that METTL3 may be a potential target for reversing chemoradiotherapy resistance.

### The role of METTL3 in glycolipid metabolism in gastrointestinal cancer

Abnormal energy metabolism (glucose metabolism, lipid metabolism, and amino acid metabolism) is one of the main characteristics of cancer [55, 60]. Cancer cells reprogram metabolism to support malignant tumor initiation and progression [61, 62]. It has been proven that the abnormal metabolism of tumor glucose and lipids is an important part of tumor metabolic reprogramming, which is closely related to tumor occurrence, development, metastasis, and recurrence [63]. In our study [33], the METTL3-HDGF axis activated glycolysis-related enzymes (ENO2 and GLUT4) at the transcriptional level to increase glycolysis, leading to GC cell proliferation and metastasis. In addition, a recent study also showed that METTL3-mediated induction of tumorigenesis in CRC was dependent upon on cell glycolysis. Mechanistically, METTL3 directly interacted with the 5′- or 3′-UTR regions of Hexokinase 2 (HK2) and the 3′-UTR region of solute carrier family 2 member 1 (GLUT1), further stabilizing these two genes and activating the glycolysis pathway [46]. Moreover, it has also been reported that a decrease in METTL3 downregulated intracellular glucose uptake and lactate production via inhibition of mTORC1 activity in HCC cells, indicating that METTL3 is involved in glycolysis activity in HCC [51]. Recent findings also suggested that increased expression of METTL3 could upregulate LINC00958 and increase lipogenesis to promote HCC progression [48]. These findings suggest that METTL3 may act as an oncogene to promote glycolysis and lipid synthesis by targeting related enzymes in gastrointestinal cancer.

### The role of METTL3 in CSCs of gastrointestinal cancer

CSCs maintain the vitality of cancer cell growth via self-renewal and infinite proliferation [64–66]. The invasion and migration of tumor stem cells make metastasis possible. CSCs can be dormant for a long time and can confer drug resistance [65, 67]. Therefore, cancer often recurs in the period of time after most common tumor cells have been eliminated by conventional therapy. Recent findings have shown that m^6^A mRNA modification is critical for the self-renewal and tumorigenesis of glioblastoma stem cells [68] and breast cancer stem cells [69]. In CRC, METTL3 facilitates CRC self-renewal and increases stem cell frequency by preventing the mRNA degradation of SOX2, a cancer stem cell marker [43]. The above results support the oncogenic role of METTL3 in promoting CRC stemness. Whether METTL3 regulates cancer stemness in other gastrointestinal cancers deserves further study.

### Upstream regulators of METTL3

**Fig. 4:**
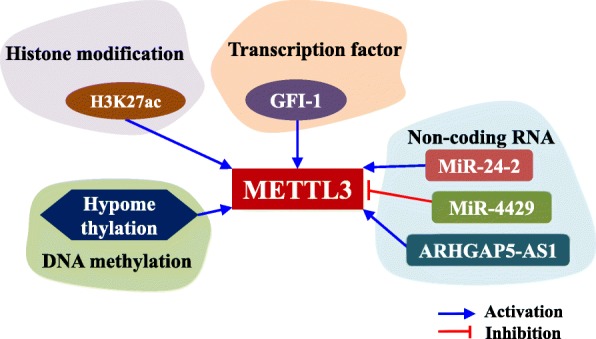
The upstream region of METTL3 in gastrointestinal cancer. Histone H3K27ac and hypomethylation at the promoter can increase METTL3 mRNA expression. The transcription factor GFI-1 might promote METTL3 mRNA expression. Non-coding RNA ARHGAP5-AS1 and miR-24-2 can promote METTL3 mRNA expression, while miR-4429 can inhibit METTL3 mRNA expression

Most studies focus on the function of METTL3 in cancer, and only a few studies have explored why METTL3 expression is abnormal in cancer. Current evidence suggests that histone modification and non-coding RNAs can influence the expression of METTL3. In GC, our study revealed that P300-mediated activation of H3K27 acetylation (H3K27ac) led to upregulated METTL3 expression in GC. In a cigarette smoke condensate-induced malignant transformation model of pancreatic duct epithelial cells, METTL3 was increased due to hypomethylation at the METTL3 promoter caused by the cigarette smoke condensate [53]. Through bioinformatics analysis, it was found that the transcription factor GFI-1 might activate METTL3 mRNA [37], but further functional verification is needed. It has been reported that miR-4429 reduces METTL3 expression in GC [36] and that miR-24-2 increases METTL3 expression in HCC (Fig. 4). Collectively, these results suggest that histone modification, promoter methylation, and non-coding RNAs can affect the expression of METTL3. Studying the upstream regulatory mechanism of METTL3 caused by epigenetic modification will allow us to better understand the biological function of METTL3 in cancers.

### Potential clinical application of METTL3

The above evidence shows that METTL3 plays a critical role in the progression of gastrointestinal cancer, indicating that METTL3 is a promising biomarker for clinical diagnosis and therapeutic response prediction and is a potential therapeutic target. According to our [33] and others’ studies [35, 40], METTL3 might be an independent prognostic factor for GC patients. We conducted a time-dependent receiver operating characteristic curve analysis, which indicated that the combination of the TNM stage and METTL3 risk score enhanced the ability of the model to predict prognosis. In addition, it has also been shown that overexpression of METTL3 contributes to a poor prognosis in CRC [43] and HCC [50] and correlates positively with tumor metastasis [41]. Therefore, METTL3 may be a biomarker of advanced GC, CRC, and HCC. Current studies are focused on the role of METTL3 in advanced gastrointestinal cancer and whether the expression of METTL3 is increased in the early stage of gastrointestinal cancer requires further study. If METTL3 is involved in the early stage of gastrointestinal cancer, it could improve early cancer diagnosis and prevention.

Given the oncogenic role of METTL3 in gastrointestinal cancer and its methyltransferase activity, it appears to be a promising therapeutic target for gastrointestinal cancer. Currently, no specific inhibitors of METTL3 have been found; thus, new potential candidates merit further exploration. Recently, Bedi et al. screened a library of 4000 analogs and derivatives of the adenosine moiety of SAM by high-throughput docking into METTL3, and two compounds showed good ligand efficiency [70]. They are further exploring potent and selective inhibitors of METTL3. Additionally, exploring inhibitors targeting molecules upstream or downstream of METTL3 is also a potential strategy for gastrointestinal cancer treatment. Chemotherapy and radiotherapy are still the main clinical treatments for gastrointestinal cancer; however, resistance to radio- and chemotherapy is inevitable and contributes to poor prognosis [59]. Therefore, the combination of chemo- and radiotherapy with inhibition of METTL3 expression or activity is a promising therapeutic strategy and is expected to be explored in the future.

## Discussion

The present review suggests that expression of the methyltransferase METTL3 is significantly increased in various gastrointestinal cancer (GC, CRC, HCC, and PC) cells compared to normal cells and acts as an oncogene to promote the progression of gastrointestinal cancer; METTL3 can serve as a clinical diagnostic and therapeutic target. However, more large-scale and multicentre data are required to further explore the function of METTL3, which may lay a foundation for individualized precision therapy in gastrointestinal cancer.

Recent findings have shown that m^6^A modification and its regulators play important and diverse biological functions in the development of various cancers [23]. Among the m^6^A modulators, METTL3 is thoroughly and widely studied [23, 71]. As noted herein, METTL3 is involved in different aspects of gastrointestinal cancer progression, including cancer cell proliferation, apoptosis, invasion, migration, metastasis, angiogenesis, radiochemotherapy resistance, glycolysis/lipid metabolism, and CSC maintenance. The underlying mechanism of METTL3 is complex and involves multiple molecules and pathways in gastrointestinal cancer (Table 1). In GC, studies by our lab and others have confirmed the oncogenic role of METTL3 in promoting the malignant process of GC by regulating different targets or pathways. Our findings revealed that METTL3 expression is significantly increased in GC tissues and correlated with poor prognosis of GC patients and that the METTL3/HDGF/GLUT4/ENO2 axis promotes GC tumorigenesis and metastasis via an increase in glycolysis and angiogenesis [33]. METTL3 also promotes GC cell growth and metastasis by interacting with LINC00470 [34], enhancing ZMYM1 [35] and SEC62 mRNA stability [36]. It has also been reported that METTL3 can activate the AKT pathway [39], MYC-mediated pathway [40], and EMT process [37] to promote cell growth and metastasis and inhibit apoptosis in GC. LncRNA ARHGAP5-AS1 recruits METTL3 to stimulate m^6^A modification of ARHGAP5 to stabilize its mRNA, resulting in chemoresistance in GC [38]. In CRC, METTL3 is highly expressed in metastatic CRC and is associated with poor prognosis [43]. METTL3-mediated m^6^A modification is closely correlated with glycolysis pathway activation in CRC patient tissues [46]. Furthermore, METTL3 promotes CRC cell growth and metastasis by promoting CRC stemness by preventing SOX2 mRNA degradation [43]. METTL3 also directly stabilizes HK2 and GLUT1 expression through an IGF2BP2/3-dependent mechanism to promote CRC tumorigenesis via an increase in glycolysis [46]. It has also been shown that METTL3 promotes the malignant process of CRC by accelerating the maturation of pri-miR-1246 [41] or stabilizing CCNE1 mRNA. METTL3 also promotes CRC multidrug resistance via the acceleration of p53 R273H pre-mRNA splicing [44]. Interestingly, only one study indicated that METTL3 is a tumor suppressor that inhibits CRC cell proliferation, migration, and invasion, which may be attributed to differences in tumor tissue origin, intratumoral heterogeneity, and research methods [42]. In HCC, METTL3 is frequently upregulated and associated with poor prognosis of HCC patients [50]. METTL3 could promote glycolysis via activation of the mTORC1 pathway and accelerate lipogenesis by enhancing LINC00958 RNA transcript stability [48], which contributes to HCC progression. In addition, METTL3 promotes the malignant process of HCC through YTHDF2-dependent silencing of SOCS2 [50] and the promotion of miR6079 expression [47]. Furthermore, METTL3 triggers polysome-mediated translation of Snail mRNA to accelerate the EMT process of HCC [49]. In PC, METTL3 promotes PC tumorigenesis by accelerating the miR-25 process and maturation [53]. It has also been reported that METTL3 can activate MAPK, ubiquitin, and RNA splicing pathways to promote chemo- and radioresistance of PC [54]. In summary, METTL3 regulates the differential expression of oncogenes and tumor suppressor genes at posttranscriptional levels by influencing their RNA stability and translation, which contribute to the development of gastrointestinal cancer.

The present review showed that METTL3 influences the progression of gastrointestinal cancer through regulating the maturation, decay, stability, translation, and splicing of mRNA or non-coding RNA. However, little attention has been paid to the role of METTL3 in the crosstalk between cancer cells and tumor-associated fibroblasts and immune cells in the tumor microenvironment. A previous study showed that the deletion of METTL3 in mouse T cells disrupts T cell homeostasis and differentiation by targeting the IL-7/STAT5/SOCS pathways [72]. Another study also showed that METTL3-mediated mRNA m^6^A methylation promotes dendritic cell (DC) activation and function. Mechanistically, METTL3-mediated m^6^A of CD40, CD80, and TLR4 signaling adaptor Tirap transcripts enhances their translation in DCs to stimulate T cell activation and promote TLR4/NF-κB signaling-induced cytokine production [73]. Interestingly, in addition to m^6^A methyltransferase activity, METTL3 also promotes the translation of target transcripts in lung cancer cells independent of its catalytic activity [74]. The molecular mechanism of METTL3 in m^6^A regulation in gastrointestinal cancer biology still needs further exploration.

Most studies focus on the downstream effects of METTL3 in the development of gastrointestinal cancer, ignoring why METTL3 expression is dysregulated in gastrointestinal cancer. The current data suggest that H3K27ac of histones and hypomethylation at promoters can increase METTL3 expression in gastrointestinal cancer [33, 53]. Non-coding RNA ARHGAP5-AS1 [38], miR-24-2 [47], and miR-4429 [36] can also regulate METTL3 expression and influence the progression of gastrointestinal cancers. Whether there are other histone modifications or non-coding RNAs involved in the regulation of METTL3 needs further research and exploration. A recent study also reported that SUMOylation of METTL3 protein does not alter its stability or localization but significantly represses its m^6^A methyltransferase activity, leading to decreased m^6^A levels on mRNAs [75]. Whether there are other post-translational modifications, such as ubiquitination and glycosylation, involved in regulating the expression of METTL3 in gastrointestinal cancer needs further study.

Current studies refer to the biological function and mechanism of METTL3. Many studies have used METTL3 as a tumor biomarker, but the specificity and sensitivity of METTL3 in different types of gastrointestinal cancer need further study. In addition, few studies have focused on the screening of METTL3 inhibitors, which have great potential, but these studies are still in the early stages.

## Conclusions

METTL3 plays a critical role in the development of gastrointestinal cancer, but there are still many problems that need further comprehensive study. Future research should be focused on (1) the role of METTL3 in the tumor microenvironment, (2) the molecular mechanism of modulating METTL3 expression and activity, and (3) the screening of specific inhibitors and their application in the clinic. Undoubtedly, METTL3 and its mediation of RNA m^6^A methylation in cancer are novel prognostic markers and predictive factors in gastrointestinal cancer.

## Data Availability

Not applicable.

## Abbreviations

ac^4^C: N4-Acetylcytidine
ALKBH5: alkB Homolog 5, RNA demethylase
ARHGAP5: Rho GTPase-activating protein 5
bcr/abl: BCR activator of RhoGEF and GTPase/ABL proto-oncogene 1, non-receptor tyrosine kinase
CCNE1: Cyclin E1
CRC: Colorectal cancer
CSC: Cancer stem cell
DC: Dendritic cell
EIF3: Eukaryotic translation initiation factor 3 subunit A
EMT: Epithelial-mesenchymal transition
ENO2: Enolase 2
FTO: FTO alpha-ketoglutarate-dependent dioxygenase
GC: Gastric cancer
GFI-1: Growth factor independence 1
GLUT1: Solute carrier family 2 member 2
GLUT4: Solute carrier family 2 member 4
H3K27ac: Histone H3K27 acetylation
HCC: Hepatocellular carcinoma
HDGF: Hepatoma-derived growth factor
HK2: Hexokinase 2
HNRNPA2B1: Heterogeneous nuclear ribonucleoprotein A2/B1
HNRNPC: Heterogeneous nuclear ribonucleoprotein C
HUVEC: Human umbilical vein endothelial cells
IGF2BP1/2/3: Insulin-like growth factor 2 mRNA-binding protein 1/2/3
IL-7: Interleukin 7
KIAA1429: VIRMA, vir-like m6A methyltransferase associated
LC: Liver cancer
m^1^A: N1-Methyladenosine
m^1^G: 1-Methylguanosine
m^2^G: 1-Methylguanosine
m^5^C: 5-Methylcytosine
m^6^A: N6-Methyladenosine
m^6^G: 1-Methylguanosine
m^7^G: 1-Methylguanosine
MDM2: MDM2 proto-oncogene
METTL14: Methyltransferase-like 14
METTL16: Methyltransferase-like 16
METTL3: Methyltransferase-like 3
MYC: MYC proto-oncogene, bHLH transcription factor
NF-κB: Nuclear factor kappa B subunit 1
P53: P53 tumor suppressor
p53: Tumor protein p53
PC: Pancreatic cancer
PTEN: Phosphatase and tensin homolog
Rb: RB transcriptional corepressor 1
RBM15: RNA-binding motif protein 15
SAM: *S*-Adenosylmethionine
SEC62: SEC62 homolog, preprotein translocation factor
Snail: Snail family transcriptional repressor
SOCS: Cytokine-inducible SH2-containing protein
SOCS2: Suppressor of cytokine signaling 2
SOX2: SRY-box 2
SPRED2: Sprouty-related EVH1 domain-containing 2
STAT5: Signal transducer and activator of transcription 5
TLR4: Toll-like receptor 4
WTAP: WT1-associated protein
YTHDC1/2: YTH domain-containing protein 1/2
YTHDF1/2/3: YTH N6-methyladenosine RNA-binding protein 1/2/3
ZC3H13: Zinc finger CCCH-type containing 13
ZMYM1: Zinc finger MYM-type containing 1
